# Uncertainty Estimation for the Brillouin Frequency Shift Measurement Using a Scanning Tandem Fabry–Pérot Interferometer

**DOI:** 10.3390/mi14071429

**Published:** 2023-07-15

**Authors:** Patrice Salzenstein, Thomas Y. Wu

**Affiliations:** 1 Centre National de la Recherche Scientifique (CNRS), Franche-Comté Electronique Mécanique Thermique Optique Sciences et Technologies (FEMTO-ST) Institute, Université de Franche-Comté (UFC), 25030 Besançon, France; 2 National Metrology Centre (NMC), Agency for Science, Technology and Research (A*STAR), 8 CleanTech Loop, #01-20, Singapore 637145, Singapore

**Keywords:** brillouin light scattering, high-power laser, tandem Fabry–Pérot interferometer, Brillouin spectroscopy, elastic property, speed of sound, measurement uncertainty analysis

## Abstract

The expanded uncertainty of the measured Brillouin scattering shift frequencies is essential in assessing the measurements of parameters of various materials. We describe the general operation principles of a Brillouin light scattering (BLS) spectrometer with a high-power laser and a scanning tandem Fabry–Pérot interferometer (TFPI) for material characterization. Various uncertainty components have been analyzed for the BLS spectrometer following the Guide to the Expression of Uncertainty in Measurement (GUM). The expanded relative uncertainty in the measured Brillouin frequency shift of 15.70 GHz for polymethyl methacrylate (PMMA) was estimated to be 0.26%. The calculated Brillouin frequency shift (based on material properties of PMMA) was determined to be 15.44 GHz with expanded relative uncertainty of 2.13%. It was shown that the measured and calculated Brillouin frequency shifts for PMMA agree within their expanded uncertainties. The TFPI-based BLS spectrometer can be used to measure the longitudinal modulus of materials with an expanded uncertainty of 1.9%, which is smaller than that of the ultrasonic velocity-based method (estimated to be 2.9%).

## 1. Introduction

The Brillouin light scattering (BLS) technique is a non-contact, non-destructive method for studying the elastic properties of materials, and it is gradually gaining popularity in various industrial applications and research laboratories. BLS is the inelastic scattering of light (photons) by thermally generated acoustic vibrations (acoustic phonons) [1,2,3]. The thermally excited sound waves in materials have very weak intensity, which cannot be detected by ultrasonic methods. We would like to recall the main contributions to the detection of sound waves, by the brothers Curie [4], and the theoretical prediction of inelastic light scattering in materials by thermally excited acoustic phonons, by Brillouin [1] and Mandelstam [2] in the 1920s. The first BLS spectrum from acoustic phonons of liquids was observed and reported in 1930 by Gross [5,6,7]. BLS measurements were reported in [8,9,10] in the 1930s. Grimsditch and Ramdas made precise measurements with Brillouin scattering on diamonds in 1975 [11].

Kojima showed that BLS techniques are very useful for studying material properties [12]. Magnons can be divided into surface-like excitations and bulk-like excitations [3,13]. The magnetic properties of materials can also be measured via their magnetic excitations (magnons) using Brillouin scattering [13]. Brillouin spectroscopy has become an essential tool for the study of acoustic phonons, magnons and the elastic properties of materials [12,14,15,16]. Brillouin spectroscopy and microscopy have emerged as non-destructive, non-contact and label-free methods for probing the viscoelastic properties of biological samples [17,18,19,20]. Brillouin scattering has also been used in atmospheric aerosol measurements, the study of air molecules and the profiling of aerosols’ optical properties [21,22,23,24,25,26,27,28].

Both Raman scattering and Brillouin scattering arise from the inelastic scattering of light. Raman scattering is associated with the scattering by optical phonons and molecular vibrations [29,30]. Brillouin spectroscopy is complementary to Raman spectroscopy in material characterization [31]. However, there are some key differences between the two types of inelastic light-scattering spectroscopy. Raman spectroscopy is based on scattering by optical phonons with frequency shifts at the THz range, and it determines the sample’s chemical composition and molecular structure using an interferometer or a dispersive spectrometer, whereas the Brillouin spectroscopy is based on scattering of acoustic phonons with frequency shifts at the GHz range, and it traditionally measures the elastic properties of materials using scanning tandem Fabry–Pérot interferometer (TFPI) [32,33,34,35,36,37,38]. Brillouin and Raman micro-spectroscopy have been combined to obtain the Brillouin and Raman spectra of biological samples simultaneously to assign their chemical specificity to mechanical properties [39,40,41]. The multimodal micro-spectroscopy developed in [42] was based on simultaneous detection of Brillouin and Raman scattering with spectral coverage of up to 100 THz.

The measurement of Brillouin spectra requires appropriate instruments such as the Fabry–Pérot interferometer (FPI), which can provide high contrast [36,37,38,43,44,45,46,47,48,49,50,51]. A double-pass FPI was implemented by Sandercock in 1970 to detect BLS [33]. Improved methods using multi-pass TFPI were reported by Lindsay, Anderson and Sandercock [52], Dil et al. [53] and Mock et al. [54] to achieve 150-dB high-contrast measurement of the Brillouin frequency shift. The scanning multi-pass TFPI technique has been further improved with automatic computer control [50,55].

The scanning TFPI requires a long acquisition time to measure the Brillouin spectra. This long acquisition time makes the traditional scanning TFPI unsuitable for high-throughput biomedical applications or dynamic measurement. To perform a rapid spectrum measurement within 1 s, a non-scanning angular dispersive FPI (ADFPI) was developed, using a solid etalon and a multichannel detector [56,57,58]. A virtually imaged phased array (VIPA) was proposed to achieve large angular dispersion [59], which can be used to build another type of ADFPI. A non-scanning Brillouin spectrometer employing a VIPA etalon and CCD camera was developed to acquire Brillouin spectra within only a few seconds [60]. Cascading three-stage VIPA etalons can provide an extinction ratio of up to 80 dB to reduce the Rayleigh scattering background and crosstalk substantially [61]. To further suppress the high scattering background, molecular or atomic absorption cells were introduced as notch filters before single-stage VIPA spectrometer to absorb the Rayleigh scattering [39,62]. In such a design, the Brillouin peak position and shape could be altered by the atomic–molecular absorption filters; therefore, a customized least-squares fitting algorithm had been proposed to retrieve the Brillouin shifts and linewidths with high accuracy [63].

Coker et al. [64] compared two VIPA-based spectrometers (780-nm and 532-nm wavelengths) with a molecular or atomic absorption cell to a scanning six-pass TFPI to assess their measurement accuracy. With an acquisition time of ~0.5 s, the three Brillouin spectrometers were used to measure the Brillouin frequency and linewidth for acetone. The results showed that the scanning TFPI yielded a smaller deviation in Brillouin frequency (from the theoretical value) and a narrower linewidth. This study explored the possibility of reducing the frequency and linewidth measurement standard deviations by extending the acquisition times using TFPI- and VIPA-based spectrometers. The authors showed that the 780-nm VIPA-based spectrometer can achieve the minimum standard deviation in Brillouin frequency and linewidth measurement (for acetone) using a much shorter acquisition time than TFPI, and its linewidth measurement accuracy was decided by the laser stability and optical components’ quality [64].

Stimulated Brillouin scattering (SBS) is a nonlinear process that has been applied in optical fibers and optoelectronic engineering [65,66,67,68]. SBS manifests itself through the creation of a backward propagating Stokes wave. Most of the input power is carried by the Stokes wave when the Brillouin threshold is reached. Impulsive and frequency-domain SBS spectroscopy and imaging systems have been developed to avoid the issue of strong Rayleigh scattering background and achieve high-speed microscopic imaging [69,70,71,72]. Ballmann et al. [73] compared the Brillouin shift measurement accuracy of impulsive SBS versus a 780-nm VIPA spectrometer, and they showed that the Allan variances for acetone measurement using impulsive SBS are much lower and decrease to the minimum values using a much shorter acquisition time. This study showed that impulsive SBS is superior to VIPA spectrometer in terms of Brillouin frequency measurement stability since the measured frequency is independent of the frequency of the pump or the probe laser.

In this paper, we show the main steps in using a scanning six-pass TFPI [74,75] to measure the Brillouin frequency shift and estimate the speed of propagation of phononic waves in materials. The objective of this paper is to assess the uncertainties in Brillouin frequency shift measurement using a TFPI via a consistent metrological approach. The measurement experiment is described in Section 2, with the results provided in Section 3. The various measurement uncertainty components associated with the Brillouin frequency shift measurement are derived in Section 4. In Section 5, we perform a comparison of the measured and calculated Brillouin frequency shifts for the test material. The Brillouin frequency shift can be used to estimate the speed of the corresponding phononic wave and elastic modulus of materials with high accuracy. The measurement uncertainty in a material’s longitudinal modulus derived by the TFPI is also reported. Finally, we discuss the limitations, potential solutions and future work in Brillouin spectroscopy in Section 6.

## 2. Materials and Methods

We present a method for the detection of BLS from a material under testing, i.e., the device under test (DUT). This material can be isotropic or anisotropic. One of the key instruments in the measurement system is the scanning TFPI. Detected spectrum peaks are shifted from the frequency of the incident laser. Those offset frequencies depend on the properties of the material of the DUT. The measured Brillouin frequency shift can be used to estimate the parameters of the material, such as the phase velocity of transverse and longitudinal waves and elastic modulus.

**Figure 1:**
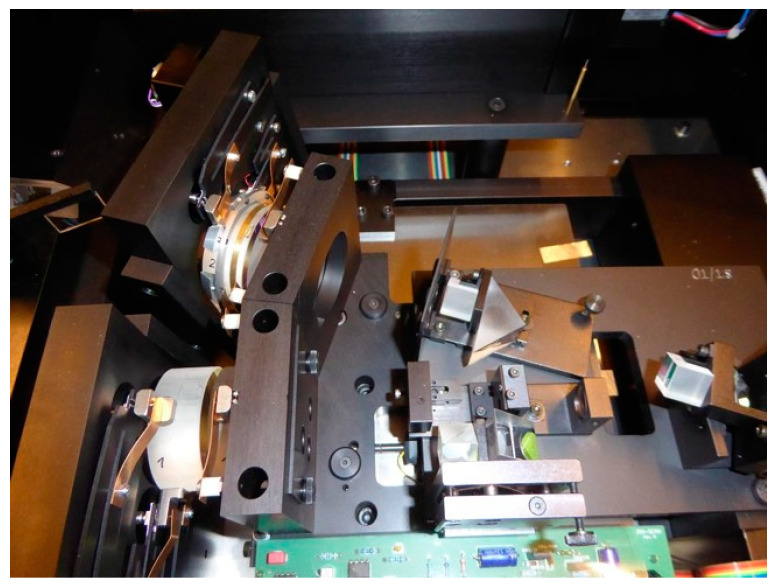
Photograph of the TFP-2, a scanning six-pass tandem Fabry–Pérot interferometer (TFPI) used for the Brillouin light scattering (BLS) measurement.

A BLS spectrometer using a 532-nm powerful Class 4 laser (up to 600 mW) is efficient to reveal the spin wave or acoustic signals at frequencies from a few gigahertz to more than 100 GHz. Fluctuations of the refractive index in a medium enables the detection and analysis of scattered laser light with the BLS spectrometer [52,53]. The TFPI (TFP-2, The Table Stable Ltd., Mettmenstetten, Switzerland [74,75]) is shown in Figure 1. The general principle is to send the signal generated by the laser and focus it on the sample that we want to characterize. The photons arrive in the material or the thin layer, and they interact with the lattice or material.

Light helps to create phonons in the material. These phonons propagate with speeds that may be different depending on whether the mode is transverse or longitudinal. The speed depends on the nature of the material, which can be isotropic or anisotropic. The phonons in turn create light, which is shifted in frequency relatively to the frequency of the incident laser. The BLS spectrometer precisely analyzes the light scattered by a material [52,55].

**Figure 2:**
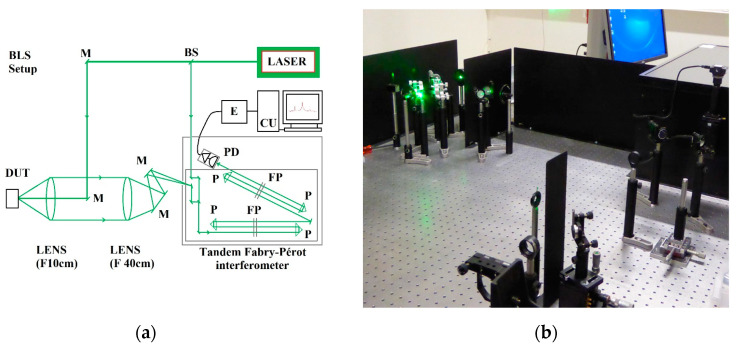
(**a**): Typical setup for BLS spectrometer (a backward scattering configuration). The TFP-2 is a commercial TPFI developed by the Table Stable Ltd. DUT: device under test. M: mirror. FP: Fabry–Pérot interferometer. P: prism. PD: photodetector. E: electronics. CU: computer unit. (**b**): The commercial TFP-2 is inside the box on the right side of this picture.

The TFPI produces spectrum peaks that are shifted from the frequency of the incident laser depending on the material. Figure 2 provides the typical setup used for the measurement, showing a backward scattering configuration (scattering angle θ = 180°) and a picture of the measurement system.

We calibrated the BLS spectrometer with part of the laser signal, used as the bench reference. Inside the commercial TFPI spectrometer (TFP-2), the light goes through two different Fabry–Pérot interferometers with six passes. Each pair of mirrors is precisely aligned during the calibration procedure.

It is necessary to calibrate the instrument accurately because it is sensitive to mechanical vibrations, temperature and humidity. The alignment process requires alignment of the two cavities. Each of the two cavities consists of a pair of parallel mirrors. TFPI produces two series of absorption peaks with respect to a flat, noisy intensity level. We then obtain a curve providing the number of absorbed photons versus frequency.

## 3. Measurement Results

Experiments can reproduce known Brillouin light scattering peaks of some bulk materials and thin films. Typical Brillouin scattering stimulations reveal acoustic or spin waves frequencies in the range between 3 and 150 GHz (though generally limited to around 30 GHz). In this section, we provide an example of a BLS spectrum with the number of detected photons versus the frequency shift for an isotropic material.

We measured a bulk material, polymethyl methacrylate (PMMA), using the BLS spectrometer. This isotropic material exhibits well-defined Brillouin frequency shift peaks. The BLS peaks are produced by sound waves in materials, and they can be analyzed by means of a damped harmonic oscillator function (DHO). From the BLS, we can deduce parameters of the material, such as the phase velocity of longitudinal waves. Knowing the *n* (optical refractive index of the material), *λ*_0_ (laser wavelength), *θ* (scattering angle) and *v* (phase velocity of longitudinal waves), the Brillouin frequency shift *ν_B_* can also be calculated by:

(1) $$\nu_{B}=\frac{{2nv}_{}}{\lambda_{0}}\;sin\;(\;\frac{ϴ}{2}\;)$$

where the phase velocity of longitudinal waves in the material can be obtained from literature or as *v* =$\sqrt{c_{11}/\rho }$, *ρ* is the density of the material and *c*_11_ is the longitudinal modulus.

**Figure 3:**
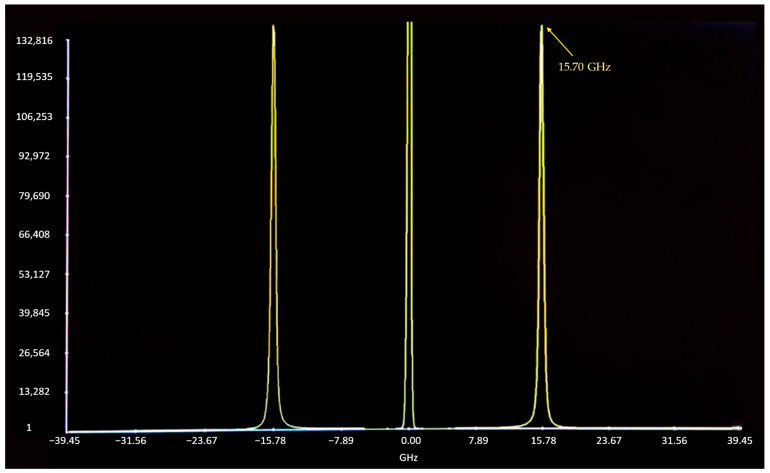
BLS spectrum for polymethyl methacrylate (PMMA) with a measured Brillouin frequency shift at 15.70 GHz (longitudinal acoustic mode, with a Brillouin linewidth of 324 MHz). Frequency shift is expressed in GHz on the horizontal axis. Vertical axis shows the number of detected photons.

We measured the *ν_B_* for PMMA as an example of an isotropic material, as shown by the BLS spectrum in Figure 3. The measured Brillouin frequency shift is *ν_B_* = 15.70 GHz (longitudinal acoustic mode), with a Brillouin linewidth of 324 MHz. The measured spectrum for PMMA (backward scattering) is shown in Figure 3. Based on the measured frequency shift *ν_B_*, the phase velocity of longitudinal waves in the test material can be derived as,

(2) $$v=\frac{\nu_{B}\lambda_{0}}{2n\;sin(\frac{ϴ}{2})}$$

With *λ*_0_ = 532 nm, *n* = 1.4953 for PMMA [76] and *θ* = 180°, the phase velocity of longitudinal waves is derived as *v* = 2792.9 m/s.

Note that, for anisotropic materials, such as sapphire, the frequency shift peaks will depend on the orientation of the DUT sample to be measured. In this case, it would be useful to check the slowness curves in the wave–vector space corresponding to the orientation of the sample with respect to the incident direction of the laser signal sent to the DUT.

## 4. Measurement Uncertainty of the Brillouin Frequency Shift

In this section, we aim to estimate the uncertainty of the Brillouin frequency shift measured by the scanning six-pass TFPI. In the scientific community, it is important to underline that a debate exists regarding whether there is a true value of the measurand. Von Clarmann et al. offered a critical discussion of the error concept versus the uncertainty concept [77]. Lee et al. [78] compared the realist view of true value measurements and its uncertainty versus the instrumentalist view of measurement (quantities are not natural attributes of the world). They showed that we need to understand the two views, and it is critical to follow the Guide to the Expression of Uncertainty in Measurement (GUM) [79].

Estimation of the measurement uncertainty requires careful analysis of the contributions from various error sources. We followed the modern way of performing the estimation of uncertainty [80]. We used a method similar to those in optical metrology [81,82,83], microwave metrology [84,85] and aerosol metrology [86,87], based on the GUM provided by the Bureau International des Poids et Mesures (BIPM) in [79]. The measurement uncertainties consist of several components, which are grouped into two main categories. These relative uncertainty terms have been normalized by the measured Brillouin frequency shift for PMMA (15.70 GHz).

### 4.1. Contributions Evaluated by Statistical Methods

Following the GUM guidelines, the first category is called type A uncertainties. It corresponds to uncertainty contributions evaluated by statistical methods, such as reproducibility and repeatability. The repeatability (denoted by A_1_) is used to show the variations in measurements obtained by one person on the same test item using the same procedure and under the same conditions (repeated in a short period of time). The repeatability for the measured Brillouin frequency shift of 15.70 GHz for PMMA is estimated to be A_1_ = 6.64 × 10^−5^.

The same operator performed the measurements, with no changes in operator behavior. All components and devices were dedicated to the BLS spectrometer, and none of them changed. Thus, the reproducibility term A_2_ can be assumed to be zero.

### 4.2. Contributions Evaluated by Other Means

The second family of uncertainty contributions consists of those assessed by other means. They are called type B uncertainties and depend on various measurement system components and ambient conditions. They are determined by the theoretical calculation, experimental experiences, general knowledge of the behavior, properties of relevant materials or measurement instrument specifications.

Frequency references of the 5-MHz or 10-MHz type ensure the frequency traceability of the BLS measurement system to national metrology standards [88,89]. It is then possible to have the best reference in terms of frequency stability to connect them to additional measuring devices, such as oscilloscopes or other frequency measurement instruments.

When there are no instrument calibration certificates, we can refer to manufacturers’ specifications, calibration data or other certificates or measurement uncertainty assigned to reference data from handbooks. Such an uncertainty term is denoted by B_R_. The BLS spectrometer was not calibrated by other metrology standards, as the method is intrinsic. Therefore, the data provided by calibration are not applicable, and we can assume that the uncertainty term B_R_ = 0.

**Figure 4:**
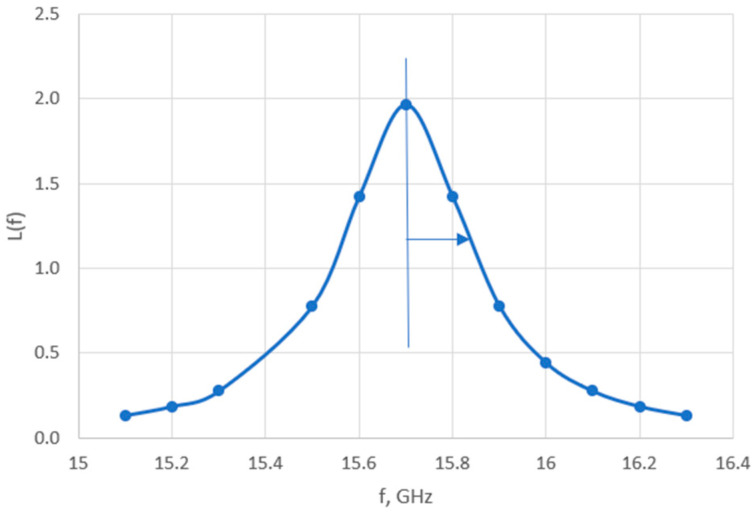
The Lorentzian function for a peak in the BLS spectrum for PMMA, assuming f_0_ = 15.70 GHz and γ = 0.16 GHz. The frequency shift is expressed in GHz on the horizontal axis.

The frequency resolution of the measurement system depends on the number of samples, i.e., the difference between two measurement frequency points along the horizontal axis. We used 2048 samples in a frequency span from 0 to 30 GHz in the BLS spectrum measurement. Thus, we have a frequency sampling interval of 14.66 MHz. The characteristic peak of Brillouin scattering has a Lorentzian distribution [90], also known as a Cauchy distribution (a probability density function). The Lorentzian function versus the frequency shift of the optical signal is given in the following expression:

(3) $$L\left(f\right)=\left(\frac{\gamma }{\pi }\right)*\left[\frac{1}{\gamma^{2}+{\left(f-f_{0}\right)}^{2}}\right]=\left(\frac{1}{\pi \gamma }\right)*\;\frac{1}{1+{\left(f-f_{0}\right)}^{2}\;{/\gamma }^{2}}\;$$

where γ is half of the frequency width at half maximum (FWHM): γ = FWHM/2; and f_0_ is the assumed true value for the Brillouin frequency shift. Figure 4 shows the Lorentzian function for a peak in the BLS spectrum for PMMA, assuming f_0_ = 15.70 GHz and γ = 0.16 GHz.

For a given true value with a peak of great smoothness, we have three points (in the worst configuration), which allow us to approximate a curve in the form of a Lorentzian function. BLS on PMMA shows well-defined peaks for isotropic materials. The maximum relative frequency error caused by the resolution limitation is (14.66/2)/15,700 = 0.00047. Assuming a rectangular distribution, the standard uncertainty due to frequency resolution is estimated as BL_0_ = 0.00047/√3 = 2.7 × 10^−4^. We can see the impact of the resolution on the uncertainty of a measured peak. There is also a risk of not detecting a peak if the sampling frequency is too low.

The uncertainty contribution of the alignment of Torus 532-nm laser (Laser Quantum Ltd., Stockport, UK) includes mainly the uncertainty due to the geometrical error and the Abbe error [91,92]. According to the manufacturer’s datasheet, the laser beam diameter is 1.7 ± 0.2 mm, the pointing direction’s stability is less than 2 µrad/°C, and the beam angle is less than 1 mrad. There is a geometrical error in the double Fabry–Pérot interferometer since some cosine error can occur. The laser beam and the axis of displacement are not completely parallel [93,94]. If we denote the angle between the two axes (beam axis and displacement axis) as A, we have an elementary term of error e_A_ = L(cosA − 1) ≈ −LA^2^/2 as A << 1. For a 1-mm distance, A is up to 10^−4^, and the relative error |e_A_/L| is up to 5 × 10^−9^, which is negligible.

The Abbe error corresponds to the magnification of angular error over distance [91,92]. The relative Brillouin frequency measurement error is proportional to the displacement error in TFPI [95]. The Abbe error is typically estimated to be about 1 nm for a Fabry–Pérot interferometer setup, which does not depend on the displacement [95]. With our BLS spectrometer, the mirror displacement range (scanning range) is up to 2.5 μm for the TFP-2. Thus, the relative Abbe error (or relative frequency measurement error) is up to 0.001/2.5 = 0.0004. This elementary term of Abbe error is a dominating term for errors caused by parallelism. Assuming a rectangular distribution for this error, the standard uncertainty in frequency shift due to Abbe error is estimated as BL_1_ = 0.0004/1.732 = 2.31 × 10^−4^.

The contribution of the laser to the noise is denoted as BL_2_. The relative intensity noise (RIN) of the laser is defined as the ratio of the average of the square of the fluctuation I optical power (δφ) to the square of the average optical power φ_0_:

RIN(ω) = <│δφ│^2^ > /φ_0_^2^ (4)

where ω is the angular frequency offset. The RIN generally presents a noise floor until the Fourier frequency, which is equal to the relaxation frequency of the laser. Beyond that frequency, the RIN decreases. This relaxation frequency is generally in the range of 1 MHz. Using a Fabry–Pérot interferometer (JRS Scientific Instruments, Mettmenstetten, Switzerland), the Torus 532-nm laser (Laser Quantum Ltd., Stockport, UK) typically shows high spectral purity with side bands <−110 dB compared with the central mode. This laser is set to operate in normal conditions between 15 °C and 35 °C. The datasheet of the Torus 532-nm laser indicates an RIN not worse than −125 dB around the frequency offset of 16 GHz. The RIN noise in the BLS spectrum will cause the peak position to have a small shift of ∆f, which can be estimated using (3). This relative frequency error is derived to be up to 8.2 × 10^−9^. We have estimated the standard uncertainty contribution due to laser’s RIN as BL_2_ = 8.2 × 10^−9^/1.732 = 4.74 × 10^−9^, assuming a rectangular distribution.

The uncertainty contribution of the photodetector Hamamatsu H10682-210 (Hamamatsu Photonics K.K., Hamamatsu City, Japan) in our BLS spectrometer is denoted as BL_3_. The datasheet of the Hamamatsu H10682-210 indicates that the specification for photon counting sensitivity is typically 4.6 × 10^5^ and 1.3 × 10^5^ s^−1^ pW^−1^ at wavelengths of 500 nm and 600 nm, respectively (5 °C to 40 °C). We can assume that it does not affect photon detection during BLS measurements. This contribution has negligible effects on the Brillouin frequency shift. Thus, the uncertainty contribution BL_3_ ≈ 0.

We have considered the uncertainty contribution of the ambient temperature, denoted as BL_4_. Temperature variation in the laboratory is in the range of 23 ± 2 °C, with maximum variation of ± 2 °C. It has been shown that a photomultiplier can have variation of 0.33% in detected peak power for a 1 °C change in temperature [96]. We assume the temperature change is within 1 °C during the BLS measurement. Thus, the 1 °C temperature change has an influence on the BLS spectrum, which is estimated to be a fluctuation of up to e_Temp_ = 10 × Log (0.9934) = −0.029 dB. This fluctuation will cause the BLS peak position to have a relative shift error of up to 8.41 × 10^−4^. The probability distribution of this error is assumed to be rectangular, and we can derive the standard uncertainty due to ambient temperature variation as BL_4_ = 8.41 × 10^−4^/1.732 = 4.86 × 10^−4^.

There is an uncertainty contribution to the laser wavelength, which is due to environmental conditions, such as ambient pressure and humidity. We denote it as BL_5_. Under normal laboratory measurement conditions, the contributions of small pressure variations and relative humidity remain negligible. Our BLS measurements do not show any dependence on those changes. Thus, this uncertainty term BL_5_ is considered to be negligible.

The uncertainty contribution due to the resolution of the power meter is denoted as BL_6_ with a rectangular distribution. It is determined by the voltmeter resolution and the value read on each voltmeter for the power meter. The maximum relative error in the frequency shift due to the voltmeter resolution is estimated be 5 × 10^−7^, which is derived assuming a spectrum noise sideband effect (−89.3 dB). Thus, the standard uncertainty due to the resolution of the power meter is derived as BL_6_ = 5 × 10^−7^/√3 = 2.89 × 10^−7^, assuming a rectangular distribution.

The uncertainty contribution of the linearity error in the scanning of the Fabry–Pérot interferometer (denoted as BL_7_) is a dominating uncertainty term in the Brillouin frequency shift [97,98]. The scan control electronics in the TFPI (for automatic scanning stage control) can produce a linearity error in the mirror spacing, which will lead to a frequency shift error. Based on the specifications of TFP-2, the linearity error is up to 0.2% [75]. Thus, the effect of the linearity error will cause a relative frequency shift error of up to 2.0 × 10^−3^. The standard uncertainty due to linearity error in scanning is derived as BL_7_ = 0.002/√3 = 1.15 × 10^−3^.

We have considered the uncertainty contributions of vibrations from the environment, which are denoted as BL_8_. We do not operate the system if there is a known vibration source in the environment. The optical table is robust enough to prevent diffusion of vibrations. Pneumatic legs are used to support the optical table. The TFPI spectrometer’s operation is only isolated against the building’s vibrations and not against vibrations introduced directly into the table. We operated the TFPI spectrometer in safe conditions and avoided any potential vibrations due to components on the table. Therefore, this uncertainty term can be assumed to be negligible.

### 4.3. Estimation of the Expanded Measurement Uncertainty

All of the uncertainty terms in measured the Brillouin frequency shift are reported in Table 1. The expanded uncertainty for the measured frequency shift with approximately 95% confidence (coverage factor *k* = 2) is calculated as follows:

(5) $$U_{m}=2\sqrt{A_{1}^{2}+A_{2}^{2}+B_{R}^{2}+\sum_{i}({BL}_{i})2}$$

From (5), the expanded relative uncertainty of the Brillouin frequency shift measurement is estimated to be U_m_ = 0.26%. For a Brillouin frequency shift measured at 15.70 GHz for PMMA, the expanded frequency measurement uncertainty is 41 MHz.

The corresponding propagation speed of the longitudinal phononic wave in PMMA can be estimated using the BLS spectrometer. Based on *v* = *ν_B_ λ*/(2*n*), the measured Brillouin frequency shift (15.70 GHz) and the refractive index of PMMA (*n =* 1.4953) [76], the longitudinal phononic wave speed in PMMA can be derived as *v* = 2792.9 m/s with an expanded uncertainty of 12.0 m/s.

## 5. Comparison with Calculated Brillouin Frequency Shift

### 5.1. Uncertainty of the Calculated Frequency Shift

For BLS measurement of PMMA, the shift frequency has been theoretically calculated using $\nu_{c}=\frac{\;2\;n\;v}{{\;\lambda }_{0}\;}\;sin(\frac{ϴ}{2})$ to be *ν_c_* = 15.44 GHz, assuming the following parameters: *n =* 1.4953 (optical refractive index of PMMA [76]), *λ*_0_ = 532 nm (laser wavelength), *θ* = 180° (scattering angle) and longitudinal phononic wave speed *v* = 2746.3 m/s (based on measurements reported in [99]).

Considering the mean bulk density of PMMA of *ρ =* 1180 kg/m^3^ (with maximum error of ± 20 kg/m^3^ [100]) and the longitudinal phononic wave speed (*v* = 2746.3 m/s) as measured in [99], the longitudinal modulus of PMMA can be estimated to be *c*_11_ = 8.8 GPa.

The longitudinal ultrasonic-velocity in PMMA was measured with repeatability of *u*_1_ = 0.31% based on data from [99], contributing to the uncertainty in the calculated frequency shift (as the repeatability in the Brillouin frequency shift). The longitudinal ultrasonic velocity measurements in PMMA showed an up to 1.74% difference when using the single-around or the pulse-echo method, as reported in [99]. This difference would lead to a standard uncertainty contribution to the calculated shift frequency of *u*_2_ = 1.74%/$\sqrt{3}$ = 1.0% (due to variability in speed measurements). The refractive index of PMMA has a maximum error of 0.3% based on the various measurement methods and data reported in [101], leading to standard uncertainty of *u*_3_ = 0.3%/$\sqrt{3}$ = 0.17% in the calculated shift frequency.

Considering these three uncertainty factors, the expanded relative uncertainty of the calculated Brillouin frequency shift (based on material mechanical properties) is estimated to be

(6) $$Uc=2\sqrt{\sum_{i}(u_{i})2}$$

As shown in Table 2, U_c_ = 2.13% (coverage factor *k* = 2).

### 5.2. Comparison of Measured and Calculated Brillouin Frequency Shift

The measured Brillouin frequency shift by the TFPI ($\nu_{m}=15.70\;GHz$) and the calculated Brillouin frequency shift ($\nu_{c}=15.44\;GHz$) for PMMA have a deviation of 0.26 GHz. To validate the estimated uncertainty for the measured Brillouin frequency shift and compare these two frequency shift values, the normalized error *E*_n_ [102] is derived as

(7) $$E_{n}=\frac{\left|\nu_{m}-\nu_{c}\right|}{\sqrt{{\nu_{m}^{2}U}_{m}^{2}+\nu_{c}^{2}U_{c}^{2}}}$$

where *U_m_* and *U_c_* are the expanded relative uncertainty of the measured and calculated Brillouin frequency shifts, respectively. This formula has been commonly used in proficiency testing or inter-laboratory comparisons.

As shown in Table 3, *E*_n_ is derived to be 0.8. Given *E*_n_ < 1, we can conclude that the measured Brillouin frequency shift ($\nu_{m}$) and calculated Brillouin frequency shift ($\nu_{c}$) for PMMA agree within their expanded uncertainties. This check using *E*_n_ derived from (7) serves to validate the expanded uncertainty that we have derived for the Brillouin frequency shift measurement in Section 4.

### 5.3. Uncertainty in the Longitudinal Modulus Derived by BLS Spectrometer

The BLS spectrometer can be used to measure the longitudinal modulus of material using

(8) $$c_{11}=\rho v^{2}=\rho (\frac{\nu_{B}\lambda_{0}}{2n\;}{)}^{2}\;$$

where $\nu_{B}$ is the measured frequency shift. Considering the standard uncertainty of 0.85% in the density of PMMA, the standard uncertainty of 0.17% in the refractive index of PMMA and the standard uncertainty of 0.13% in Brillouin frequency shift measurements, we have estimated the expanded uncertainty in the derived longitudinal modulus $c_{11}$ to be 1.9% (coverage factor *k* = 2) using (8).

The longitudinal modulus of materials can also be estimated using the ultrasonic velocity-based method via $c_{11}=\rho v^{2}$, where v is the measured ultrasonic velocity in the material. The standard uncertainty contribution from the longitudinal ultrasonic velocity measurement repeatability (for PMMA) is estimated to be 0.62%. The standard uncertainty in the density of PMMA can be estimated as 0.85%, and the standard uncertainty due to ultrasonic velocity measurement variability has been estimated to be 1.0%. Thus, the expanded uncertainty in $c_{11}$ using the ultrasonic velocity-based method is estimated to be 2.9% (coverage factor *k* = 2).

Comparing the expanded uncertainty in estimation of the longitudinal modulus of materials using the two methods, the BLS spectrometer-based method has a smaller expanded uncertainty of 1.9%. Thus, the Brillouin frequency shift can be measured to derive the longitudinal modulus of materials with higher accuracy compared to the ultrasonic velocity-based method.

## 6. Discussion and Conclusions

Brillouin spectroscopy is a non-intrusive measurement method for bulk materials and thin films. A scanning six-pass TFPI has been described for BLS measurement. Following the GUM, we performed detailed analysis and estimation of the uncertainties in the Brillouin frequency shift measurement related to the speed of propagation of phononic waves in bulk materials. The expanded relative uncertainty in the measured Brillouin frequency shift was estimated to be 0.26% (coverage factor *k* = 2), corresponding to an expanded uncertainty of 41 MHz for the measured frequency shift of 15.70 GHz in testing PMMA.

We have also estimated the expanded relative uncertainty of the calculated Brillouin frequency shift (at 15.44 GHz based on PMMA’s mechanical properties) to be 2.13% (*k* = 2). It was shown that the measured and calculated Brillouin frequency shifts for PMMA agree within their expanded uncertainties. The scanning six-pass TFPI can be used to measure the longitudinal modulus of materials with an expanded uncertainty of 1.9%, which is smaller than that of the ultrasonic velocity-based method (estimated to be 2.9%). In our future work, we will conduct uncertainty analysis for PMMA’s Brillouin linewidth measurements using the six-pass TFPI and assess the sample’s temperature stabilization effect.

Although the scanning TFPI has high accuracy in Brillouin frequency and linewidth measurements, it has limitations in the complex system design, high cost and slow acquisition time. Hence, a scanning TFPI is not suitable for biomedical imaging or for probing the viscoelastic properties of biological samples. VIPA-based spectrometers with very fast acquisition times have been developed for biomedical imaging, but such spontaneous Brillouin scattering-based spectrometers still face the issue of a high Rayleigh scattering background, making it difficult to detect the weak Brillouin signals. An impulsive SBS method [71] had been proposed to further reduce the acquisition time, reduce the standard deviations in BLS measurements and improve the spectral resolution of 2D biomedical imaging. However, the impulsive SBS microscopy still needs to improve its data acquisition speed (using a detector array), detection sensitivity, spatial resolution, etc.

SBS spectroscopy can provide high-intensity signals to improve the signal-to-noise ratio (SNR) in BLS measurements. One disadvantage of this method is that the high optical power could cause phototoxicity or thermal damage to biological samples. Therefore, the overall illumination dosage needs to be controlled. The detection limit of frequency-domain or impulsive SBS spectroscopy needs to be further improved.

Quantum-correlated light (squeezed light) can be used to squeeze the amplitude noise to a level below the vacuum-state (shot-noise) limit [103,104,105,106,107]. Quantum-enhanced sensing with single-mode or two-mode squeezing has been used to improve the SNR in gravitational wave detection, Raman spectroscopy, saturation spectroscopy, Raman microscopy, microparticle tracking, etc. [108,109,110,111,112,113,114]. Li et al. [115] showed that two-mode intensity-difference squeezed light (generated by the four-wave mixing process in atomic ^85^Rb vapor) can be used to improve the SNR of SBS spectroscopy by 3.4 dB. This quantum-enhanced SBS spectroscopy could still measure the Brillouin frequency and linewidth of water with good accuracy when the optical pump power was reduced to 7.5 mW. The quantum squeezed light has a narrow spectral width in the range of 10 MHz, which enables the improvement of the SNR in SBS spectroscopy. Quantum sensing using squeezed light is expected to further improve the quantum noise reduction in the future via the reduction of optical loss in the sensors, novel detection techniques for quantum squeezing and cross-correlation measurements for detecting a squeezed state [105,116,117,118,119].

Future metrology works could study how to obtain corrected Brillouin spectra, which represents the scattering intensity and linewidth of given samples, as a function of Brillouin frequency shifts and temperatures. Calibration standards would be needed to check the reproducibility of observed Brillouin frequency shifts and linewidths. Luminescent intensity reference standards would also be needed to calibrate various Brillouin spectrometers and make corrections for instrument response variations across a Brillouin spectrum for a specified temperature range.

## Figures and Tables

**Table 1:** Uncertainty budget table for Brillouin frequency shift measurement using our scanning six-pass TFPI spectrometer.

Uncertainty Component	Probability Distribution	Standard Relative Uncertainty Contribution
Repeatability	t-distribution	6.64 × 10^−5^
Reproducibility	t-distribution	Negligible
Frequency resolution	rectangular	2.69 × 10^−4^
Geometrical error in the BLS spectrometer	rectangular	Negligible
Abbe error in BLS spectrometer	rectangular	2.31 × 10^−7^
Relative intensity noise (RIN) of laser	rectangular	4.74 × 10^−9^
Photodetector’s counting sensitivity	rectangular	Negligible
Ambient temperature variation	rectangular	4.86 × 10^−4^
Ambient pressure and humidity variation	rectangular	Negligible
Resolution of voltmeter for power meter	rectangular	2.89 × 10^−7^
Linearity error in scanning of TFPI	rectangular	1.15 × 10^−3^
Vibration effect	rectangular	Negligible
**Combined relative uncertainty for frequency shift measurement**		0.13%
**Expanded relative uncertainty for frequency shift measurement (*k* = 2)**		0.26%

**Table 2:** Uncertainty budget table for calculated Brillouin frequency shift (using mechanical properties of PMMA).

Uncertainty Component	Probability Distribution	Standard Relative Uncertainty Contribution
Repeatability	t-distribution	0.31%
Refractive index of PMMA	rectangular	0.17%
Ultrasonic velocity measurement variation	rectangular	1.00%
**Combined relative uncertainty for calculated frequency shift**		1.06%
**Expanded relative uncertainty for calculated frequency shift (*k* = 2)**		2.13%

**Table 3:** Comparison of the measured Brillouin frequency shift ($\nu_{m}$) and calculated Brillouin frequency shift ($\nu_{c}$) for PMMA.

Measured Frequency Shift, GHz	Expanded Uncertainty of Measured Frequency Shift, (*ν_m_ U_m_*), GHz	Calculated Frequency Shift, GHz	Expanded Uncertainty of Calculated Frequency Shift (*ν_c_ U_c_*), GHz	Deviation of Frequency Shift, GHz	En
15.70	0.041	15.44	0.33	0.26	0.8

## Data Availability

Data sharing is not applicable to this article.

## References

[1] Brillouin L. (1922). Diffusion de La Lumière et Des Rayons X Par Un Corps Transparent Homogène. Ann. Phys..

[2] Mandelstam L. (1926). Light Scattering by Inhomogeneous Media. Zh. Russ. Fiz. Khim. Ova..

[3] Blachowicz T., Grimsditch M. (2005). Scattering, Inelastic: Brillouin. Encycl. Condens. Matter Phys..

[4] Curie J., Curie P. (1880). Développement Par Compression de l’électricité Polaire Dans Les Cristaux Hémièdres à Faces Inclinées. Bull. Minéral..

[5] Gross E. (1930). Change of Wave-Length of Light Due to Elastic Heat Waves at Scattering in Liquids. Nature.

[6] Gross E. (1930). The Splitting of Spectral Lines at Scattering of Light by Liquids. Nature.

[7] Gross E. (1930). Über Änderung der WellenlÄnge Bei Lichtzerstreuung in Kristallen. Z. Phys..

[8] Gross E. (1932). Modification of Light Quanta by Elastic Heat Oscillations in Scattering Media. Nature.

[9] Raghavendra Rao B.V. (1934). Examination of Molecularly Scattered Light with a Fabry-Perot Etalon—Part I. Liquid Benzene. Proc. Indian Acad. Sci.-Sect. A.

[10] Raghavendra Rao B.V. (1935). Examination of Molecularly Scattered Light with a Fabry-Perot Etalon—Part II. Liquids: Toluene and Carbon Tetrachloride. Proc. Indian Acad. Sci.-Sect. A.

[11] Grimsditch M.H., Ramdas A.K. (1975). Brillouin Scattering in Diamond. Phys. Rev. B.

[12] Kojima S. (2022). 100th Anniversary of Brillouin Scattering: Impact on Materials Science. Materials.

[13] Borovik-Romanov A.S., Kreines N.M. (1982). Brillouin-Mandelstam Scattering from Thermal and Excited Magnons. Phys. Rep..

[14] Kargar F., Balandin A.A. (2021). Advances in Brillouin–Mandelstam Light-Scattering Spectroscopy. Nat. Photonics.

[15] Merklein M., Kabakova I.V., Zarifi A., Eggleton B.J. (2022). 100 Years of Brillouin Scattering: Historical and Future Perspectives. Appl. Phys. Rev..

[16] Singaraju A.B., Bahl D., Stevens L.L. (2019). Brillouin Light Scattering: Development of a Near Century-Old Technique for Characterizing the Mechanical Properties of Materials. AAPS PharmSciTech.

[17] Palombo F., Fioretto D. (2019). Brillouin Light Scattering: Applications in Biomedical Sciences. Chem. Rev..

[18] Prevedel R., Diz-Muñoz A., Ruocco G., Antonacci G. (2019). Brillouin Microscopy: An Emerging Tool for Mechanobiology. Nat. Methods.

[19] Meng Z., Traverso A.J., Ballmann C.W., Troyanova-Wood M.A., Yakovlev V. (2016). V Seeing Cells in a New Light: A Renaissance of Brillouin Spectroscopy. Adv. Opt. Photon..

[20] Antonacci G., Beck T., Bilenca A., Czarske J., Elsayad K., Guck J., Kim K., Krug B., Palombo F., Prevedel R. (2020). Recent Progress and Current Opinions in Brillouin Microscopy for Life Science Applications. Biophys. Rev..

[21] Chitanvis S.M., Cantrell C.D. (1989). Simple Approach to Stimulated Brillouin Scattering in Glass Aerosols. J. Opt. Soc. Am. B.

[22] Boyarchuk K.A., Lyakhov G.A., Svirko Y.P. (1993). Microwave Driven Anti-Stokes Brillouin Scattering in an Ionized Medium. The Method of Remote Sensing of Atmospheric Aerosols. J. Phys. D. Appl. Phys..

[23] Hair J.W., Hostetler C.A., Cook A.L., Harper D.B., Ferrare R.A., Mack T.L., Welch W., Izquierdo L.R., Hovis F.E. (2008). Airborne High Spectral Resolution Lidar for Profiling Aerosol Optical Properties. Appl. Opt..

[24] Esselborn M., Wirth M., Fix A., Tesche M., Ehret G. (2008). Airborne High Spectral Resolution Lidar for Measuring Aerosol Extinction and Backscatter Coefficients. Appl. Opt..

[25] Gu Z., Witschas B., Van De Water W., Ubachs W. (2013). Rayleigh-Brillouin Scattering Profiles of Air at Different Temperatures and Pressures. Appl. Opt..

[26] Witschas B. (2012). Light Scattering on Molecules in the Atmosphere. Atmospheric Physics, Research Topics in Aerospace.

[27] Gu Z., Witschas B., Ubachs W. (2014). Temperature Retrieval from Rayleigh-Brillouin Scattering Profiles Measured in Air. Opt. Express.

[28] Rank D.H., Wiggins T.A., Wick R.V., Eastman D.P., Guenther A.H. (1966). Stimulated Brillouin Effect in High-Pressure Gases. J. Opt. Soc. Am..

[29] Raman C.V., Krishnan K.S. (1928). A New Type of Secondary Radiation. Nature.

[30] Raman C.V. (1928). A New Radiation. Indian J. Phys..

[31] Hendra P.J., Stratton P.M. (1969). Laser-Raman Spectroscopy. Chem. Rev..

[32] Sandercock J.R. (1975). Some Recent Developments in Brillouin Scattering. RCA Rev..

[33] Sandercock J.R. (1970). Brillouin Scattering Study of SbSI Using a Double-Passed, Stabilised Scanning Interferometer. Opt. Commun..

[34] Sandercock J.R., Murphy W.F. (1980). Light Scattering from Thermally Excited Surface Phonons and Magnons. 7th International Conference on Raman Spectroscopy.

[35] Sandercock J.R. (1982). Trends in Brillouin Scattering: Studies of Opaque Materials, Supported Films, and Central Modes. Topics in Applied Physics.

[36] Vacher R., Boyer L., Boissier M. (1972). Measurement of the Elastic Constants of Lithium Acetate by Means of the Brillouin Effect. Phys. Rev. B.

[37] Sussner H., Vacher R. (1979). High-Precision Measurements of Brillouin Scattering Frequencies. Appl. Opt..

[38] Vacher R., Sussner H., Schickfus M.V. (1980). A Fully Stabilized Brillouin Spectrometer with High Contrast and High Resolution. Rev. Sci. Instrum..

[39] Traverso A.J., Thompson J.V., Steelman Z.A., Meng Z., Scully M.O., Yakovlev V.V. (2015). Dual Raman-Brillouin Microscope for Chemical and Mechanical Characterization and Imaging. Anal. Chem..

[40] Mattana S., Alunni Cardinali M., Caponi S., Casagrande Pierantoni D., Corte L., Roscini L., Cardinali G., Fioretto D. (2017). High-Contrast Brillouin and Raman Micro-Spectroscopy for Simultaneous Mechanical and Chemical Investigation of Microbial Biofilms. Biophys. Chem..

[41] Meng Z., Bustamante Lopez S.C., Meissner K.E., Yakovlev V.V. (2016). Subcellular Measurements of Mechanical and Chemical Properties Using Dual Raman-Brillouin Microspectroscopy. J. Biophotonics.

[42] Scarponi F., Mattana S., Corezzi S., Caponi S., Comez L., Sassi P., Morresi A., Paolantoni M., Urbanelli L., Emiliani C. (2017). High-Performance Versatile Setup for Simultaneous Brillouin-Raman Microspectroscopy. Phys. Rev. X.

[43] Aschauer R., Asenbaum A., Geri H. (1990). Fabry-Perot Interferometer with Personal Computer Control. Appl. Opt..

[44] Błachowicz T., Bukowski R., Kleszczewski Z. (1996). Fabry-Perot Interferometer in Brillouin Scattering Experiments. Rev. Sci. Instrum..

[45] Hernandez G. (1988). Fabry-Perot Interferometers.

[46] Perot A., Fabry C. (1899). On the Application of Interference Phenomena to the Solution of Various Problems of Spectroscopy and Metrology. Astrophys. J..

[47] Fabry C., Pérot A. (1899). Théorie et Applications d’une Nouvelle Méthode de Spectroscopie Interférentielle. Ann. Chim. Phys..

[48] Heiman D., Hamilton D.S., Hellwarth R.W. (1979). Brillouin Scattering Measurements on Optical Glasses. Phys. Rev. B.

[49] Asenbaum A. (1979). Computer-Controlled Fabry-Perot Interferometer for Brillouin Spectroscopy. Appl. Opt..

[50] Salvato G., Ponterio R.C., Aliotta F. (2006). New Automatic System for Multipass Fabry-Ṕrot Alignment and Stabilization. Rev. Sci. Instrum..

[51] Błachowicz T. (2000). The Use of Pressure Controlled Fabry-Pérot Interferometer with Linear Scanning of Data for Brillouin-Type Experiments. Rev. Sci. Instrum..

[52] Lindsay S.M., Anderson M.W., Sandercock J.R. (1981). Construction and Alignment of a High Performance Multipass Vernier Tandem Fabry-Perot Interferometer. Rev. Sci. Instrum..

[53] Dil J.G., van Hijningen N.C.J.A., van Dorst F., Aarts R.M. (1981). Tandem Multipass Fabry-Perot Interferometer for Brillouin Scattering. Appl. Opt..

[54] Mock R., Hillebrands B., Sandercock R. (1987). Construction and Performance of a Brillouin Scattering Set-up Using a Triple-Pass Tandem Fabry-Perot Interferometer. J. Phys. E.

[55] Hillebrands B. (1999). Progress in Multipass Tandem Fabry-Perot Interferometry: I. A Fully Automated, Easy to Use, Self-Aligning Spectrometer with Increased Stability and Flexibility. Rev. Sci. Instrum..

[56] Itoh S.I., Yamana T., Kojima S. (1996). Quick Measurement of Brillouin Spectra of Glass-Forming Material Trimethylene Glycol by Angular Dispersion-Type Fabry-Perot Interferometer System. Jpn. J. Appl. Phys. Part 1 Regul. Pap. Short Notes Rev. Pap..

[57] Ko J.H., Kojima S. (2002). Nonscanning Brillouin Spectroscopy Applied to Solid Materials. Rev. Sci. Instrum..

[58] Ike Y., Tsukada S., Kojima S. (2007). High-Resolution Brillouin Spectroscopy with Angular Dispersion-Type Fabry-Perot Interferometer and Its Application to a Quartz Crystal. Rev. Sci. Instrum..

[59] Shirasaki M. (1996). Large Angular Dispersion by a Virtually Imaged Phased Array and Its Application to a Wavelength Demultiplexer. Opt. Lett..

[60] Scarcelli G., Yun S.H. (2008). Confocal Brillouin Microscopy for Three-Dimensional Mechanical Imaging. Nat. Photonics.

[61] Scarcelli G., Yun S.H. (2011). Multistage VIPA Etalons for High-Extinction Parallel Brillouin Spectroscopy. Opt. Express.

[62] Meng Z., Traverso A.J., Yakovlev V.V. (2014). Background Clean-up in Brillouin Microspectroscopy of Scattering Medium. Opt. Express.

[63] Meng Z., Yakovlev V.V. (2016). Precise Determination of Brillouin Scattering Spectrum Using a Virtually Imaged Phase Array (VIPA) Spectrometer and Charge-Coupled Device (CCD) Camera. Appl. Spectrosc..

[64] Coker Z., Troyanova-Wood M., Traverso A.J., Yakupov T., Utegulov Z.N., Yakovlev V.V. (2018). Assessing Performance of Modern Brillouin Spectrometers. Opt. Express.

[65] Cotter D. (1983). Stimulated Brillouin Scattering in Monomode Optical Fiber. J. Opt. Commun..

[66] Garmire E. (2018). Stimulated Brillouin Review: Invented 50 Years Ago and Applied Today. Int. J. Opt..

[67] Bai Z., Yuan H., Liu Z., Xu P., Gao Q., Williams R.J., Kitzler O., Mildren R.P., Wang Y., Lu Z. (2018). Stimulated Brillouin Scattering Materials, Experimental Design and Applications: A Review. Opt. Mater..

[68] Agrawal G.P. (2019). Stimulated Brillouin Scattering. Nonlinear Fiber Optics.

[69] Ballmann C.W., Thompson J.V., Traverso A.J., Meng Z., Scully M.O., Yakovlev V.V. (2015). Stimulated Brillouin Scattering Microscopic Imaging. Sci. Rep..

[70] Krug B., Koukourakis N., Czarske J.W. (2019). Impulsive Stimulated Brillouin Microscopy for Non-Contact, Fast Mechanical Investigations of Hydrogels. Opt. Express.

[71] Ballmann C.W., Meng Z., Traverso A.J., Scully M.O., Yakovlev V.V. (2017). Impulsive Brillouin Microscopy. Optica.

[72] Remer I., Shaashoua R., Shemesh N., Ben-Zvi A., Bilenca A. (2020). High-Sensitivity and High-Specificity Biomechanical Imaging by Stimulated Brillouin Scattering Microscopy. Nat. Methods.

[73] Ballmann C.W., Meng Z., Yakovlev V.V. (2019). Nonlinear Brillouin Spectroscopy: What Makes It a Better Tool for Biological Viscoelastic Measurements. Biomed. Opt. Express.

[74] JRS (2023). Brillouin Scattering by Means of the JRS TFP-1 Tandem Multi-Pass Fabry-Pérot Interferometer.

[75] Table Stable (2023). Tandem Fabry-Pérot Spectrometers TFP-1 and TFP-2 HC (Operator Manual).

[76] Beadie G., Brindza M., Flynn R.A., Rosenberg A., Shirk J.S. (2015). Refractive Index Measurements of Poly(Methyl Meth-Acrylate) (PMMA) from 0.4–1.6 μm. Appl. Opt..

[77] Von Clarmann T., Compernolle S., Hase F. (2022). Truth and Uncertainty. A Critical Discussion of the Error Concept versus the Uncertainty Concept. Atmos. Meas. Tech..

[78] Lee J.W., Hwang E., Kacker R.N. (2022). True Value, Error, and Measurement Uncertainty: Two Views. Accredit. Qual. Assur..

[79] (2008). Uncertainty of Measurement—Part 3: Guide to the Expression of Uncertainty in Measurement (GUM:1995).

[80] Kacker R., Sommer K.D., Kessel R. (2007). Evolution of Modern Approaches to Express Uncertainty in Measurement. Metrologia.

[81] Salzenstein P., Pavlyuchenko E., Hmima A., Cholley N., Zarubin M., Galliou S., Chembo Y.K., Larger L. (2012). Estimation of the Uncertainty for a Phase Noise Optoelectronic Metrology System. Phys. Scr..

[82] Pavlyuchenko E., Salzenstein P. Application of Modern Method of Calculating Uncertainty to Microwaves and Opto-Electronics. Proceedings of the 2014 International Conference Laser Optics.

[83] Salzenstein P., Pavlyuchenko E. (2021). Uncertainty Evaluation on a 10.52 GHz (5 dBm) Optoelectronic Oscillator Phase Noise Performance. Micromachines.

[84] Lee W.K., Yu D.H., Park C.Y., Mun J. (2010). The Uncertainty Associated with the Weighted Mean Frequency of a Phase-Stabilized Signal with White Phase Noise. Metrologia.

[85] Salzenstein P., Wu T.Y. (2016). Uncertainty Analysis for a Phase-Detector Based Phase Noise Measurement System. Meas. J. Int. Meas. Confed..

[86] Wu T.Y., Murashima Y., Sakurai H., Iida K. (2022). A Bilateral Comparison of Particle Number Concentration Standards via Calibration of an Optical Particle Counter for Number Concentration up to ~1000 cm^−3^. Measurement.

[87] Wu T.Y., Horender S., Tancev G., Vasilatou K. (2022). Evaluation of Aerosol-Spectrometer Based PM2.5 and PM10 Mass Concentration Measurement Using Ambient-like Model Aerosols in the Laboratory. Measurement.

[88] Salzenstein P., Kuna A., Sojdr L., Chauvin J. (2010). Significant Step in Ultra-High Stability Quartz Crystal Oscillators. Electron. Lett..

[89] Salzenstein P., Cholley N., Kuna A., Abbé P., Lardet-Vieudrin F., Šojdr L., Chauvin J. (2012). Distributed Amplified Ultra-Stable Signal Quartz Oscillator Based. Meas. J. Int. Meas. Confed..

[90] Gough W. (1968). The Graphical Analysis of a Lorentzian Function and a Differentiated Lorentzian Function. J. Phys. A Gen. Phys..

[91] Köning R., Flügge J., Bosse H. (2007). A Method for the in Situ Determination of Abbe Errors and Their Correction. Meas. Sci. Technol..

[92] Leach R. (2014). Abbe Error/Offset. CIRP Encycl. Prod. Eng..

[93] Howard L., Stone J., Fu J. (2001). Real-Time Displacement Measurements with a Fabry-Perot Cavity and a Diode Laser. Precis. Eng..

[94] Joo K.N., Ellis J.D., Spronck J.W., Munnig Schmidt R.H. (2009). Design of a Folded, Multi-Pass Fabry-Perot Cavity for Displacement Metrology. Meas. Sci. Technol..

[95] Zhu M., Wei H., Wu X., Li Y. (2015). Fabry–Perot Interferometer with Picometer Resolution Referenced to an Optical Frequency Comb. Opt. Lasers Eng..

[96] Frach T., Prescher G., Degenhardt C., De Gruyter R., Schmitz A., Ballizany R. The Digital Silicon Photomultiplier—Principle of Operation and Intrinsic Detector Performance. Proceedings of the IEEE Nuclear Science Symposium Conference Record.

[97] Atherton P.D. (1995). The Scanning Fabry-Perot Spectrometer. Int. Astron. Union Colloq..

[98] Lindsay S.M., Shepherd I.W. (1977). Linear Scanning Circuit for a Piezoelectrically Controlled Fabry-Perot Etalon. Rev. Sci. Instrum..

[99] Afifi H.A. (2003). Ultrasonic Pulse Echo Studies of the Physical Properties of PMMA, PS, and PVC. Polym.-Plast. Technol. Eng..

[100] Root S.E., Gao R., Abrahamsson C.K., Kodaimati M.S., Ge S., Whitesides G.M. (2021). Estimating the Density of Thin Polymeric Films Using Magnetic Levitation. ACS Nano.

[101] Polyanskiy M.N. Refractive Index Database. https://refractiveindex.info/?shelf=organic&book=poly(methyl_methacrylate).

[102] (2010). Conformity Assessment—General Requirements for Proficiency Testing.

[103] Caves C.M. (1981). Quantum-Mechanical Noise in an Interferometer. Phys. Rev. D.

[104] Slusher R.E., Hollberg L.W., Yurke B., Mertz J.C., Valley J.F. (1985). Observation of Squeezed States Generated by Four-Wave Mixing in an Optical Cavity. Phys. Rev. Lett..

[105] Lawrie B.J., Lett P.D., Marino A.M., Pooser R.C. (2019). Quantum Sensing with Squeezed Light. ACS Photonics.

[106] Giovannetti V., Lloyd S., Maccone L. (2004). Quantum-Enhanced Measurements: Beating the Standard Quantum Limit. Science.

[107] Vahlbruch H., Mehmet M., Danzmann K., Schnabel R. (2016). Detection of 15 dB Squeezed States of Light and Their Application for the Absolute Calibration of Photoelectric Quantum Efficiency. Phys. Rev. Lett..

[108] Polzik E.S., Carri J., Kimble H.J. (1992). Spectroscopy with Squeezed Light. Phys. Rev. Lett..

[109] Casacio C.A., Madsen L.S., Terrasson A., Waleed M., Barnscheidt K., Hage B., Taylor M.A., Bowen W.P. (2021). Quantum-Enhanced Nonlinear Microscopy. Nature.

[110] Yu H., McCuller L., Tse M., Kijbunchoo N., Barsotti L., Mavalvala N., Betzwieser J., Blair C.D., Dwyer S.E., Effler A. (2020). Quantum Correlations between Light and the Kilogram-Mass Mirrors of LIGO. Nature.

[111] Taylor M.A., Janousek J., Daria V., Knittel J., Hage B., Bachor H.A., Bowen W.P. (2013). Biological Measurement beyond the Quantum Limit. Nat. Photonics.

[112] Schnabel R., Mavalvala N., McClelland D.E., Lam P.K. (2010). Quantum Metrology for Gravitational Wave Astronomy. Nat. Commun..

[113] Tse M., Yu H., Kijbunchoo N., Fernandez-Galiana A., Dupej P., Barsotti L., Blair C.D., Brown D.D., Dwyer S.E., Effler A. (2019). Quantum-Enhanced Advanced LIGO Detectors in the Era of Gravitational-Wave Astronomy. Phys. Rev. Lett..

[114] de Andrade R.B., Kerdoncuff H., Berg-Sørensen K., Gehring T., Lassen M., Andersen U.L. (2020). Quantum-Enhanced Continuous-Wave Stimulated Raman Scattering Spectroscopy. Optica.

[115] Li T., Li F., Liu X., Yakovlev V.V., Agarwal G.S. (2022). Quantum-Enhanced Stimulated Brillouin Scattering Spectroscopy and Imaging. Optica.

[116] Danilishin S.L., Khalili F.Y., Miao H. (2019). Advanced Quantum Techniques for Future Gravitational-Wave Detectors. Living Rev. Relativ..

[117] Dorfman K.E., Schlawin F., Mukamel S. (2016). Nonlinear Optical Signals and Spectroscopy with Quantum Light. Rev. Mod. Phys..

[118] Li T., Anderson B.E., Horrom T., Schmittberger B.L., Jones K.M., Lett P.D. (2017). Improved Measurement of Two-Mode Quantum Correlations Using a Phase-Sensitive Amplifier. Opt. Express.

[119] Taylor M.A., Bowen W.P. (2016). Quantum Metrology and Its Application in Biology. Phys. Rep..

